# Evolution of the physical characteristics of the French women's rugby players: A 10-year longitudinal analysis by position and team

**DOI:** 10.3389/fspor.2023.1120162

**Published:** 2023-04-05

**Authors:** Sébastien Imbert, Julien Piscione, Anthony Couderc, Hélène Joncheray, Frédéric N. Daussin

**Affiliations:** ^1^ Univ. Lille, Univ. Artois, Univ. Littoral Côte d’Opale, ULR 7369—URePSSS—Unité de Recherche Pluridisciplinaire Sport Santé Société, Lille, France; ^2^ Ligue des Hauts-de-France de Rugby, Villeneuve D’ascq, France; ^3^ Department of Performance, Fédération Française de Rugby, Marcoussis, France; ^4^ Laboratoire Sport, Expertise, Performance, Unité de la Recherche, Institut National du Sport, de L’Expertise et de la Performance (INSEP), Paris, France

**Keywords:** women, rugby, performance, anthropometric parameters, strength, speed, aerobic fitness

## Abstract

**Introduction:**

The study aimed to interpret the evolution of the physical performance of rugby sevens and rugby union French international players from 2009 to 2020.

**Methods:**

631 players from the French national teams were divided into three groups: forwards, backs and sevens. The performances evaluated were anthropometric characteristics, strength tests (1 RM bench press and 1 RM pull-up), aerobic capacity (YoYo IR1 test) and speed tests (10 m, 20 m and 50 m). The best performance of each player over a two-year period was kept for the analysis. Fluctuations were observed across the decade.

**Results:**

The anthropometric characteristics of female rugby sevens players tend to be taller and lighter than rugby union players. In rugby sevens, a moderate increase in maximal aerobic capacity was observed while sprint performances remained similar. Improvements in height and weight were observed over the last 10 years in rugby union players with a difference between the position. A moderate increase in sprinting performances and strength were observed both in backs and forwards.

**Discussion:**

The overall improvement of strength and conditioning performances and anthropometrical evolution reflects the rugby environment characterized by the arrival of professional contracts and the structuration process of the clubs which allows a better quality of training and easier access to the infrastructures of the very high level.

## 1. Introduction

Rugby is a team sport that is characterized by a large number of high-impact collisions interspaced with maximal sprints, and the aim is to score points by hand and/or foot by flattening the ball in the opponent's in-goal or by passing it between the posts (1). The two major disciplines played in France are rugby union and sevens, which displays differences in the length of matches (14 min vs. 80 min, respectively, for sevens and rugby union) and the number of players (7 vs. 15 players, respectively, for sevens and rugby union) (2–5) but the types of collisions are similar (i.e., tackles, scrums, rucks, and mauls) (5). Currently, and as opposed to male rugby, there is a paucity in the literature about the physical demands of rugby union and rugby sevens' matches, which has been raised as a current concern in female sport (6, 7). Such information is of interest to determine the best training programs and performance outputs for recruitment and player development (8, 9).

Previous studies provided initial insight into the anthropometric and physical characteristics of elite female rugby players (10, 11). Recently, it has been described that professional female rugby union players exhibit significant anthropometric and physical differences among the playing positions (12). Most of the studies focus on physical demands, which increased over time, as depicted in a 5-year longitudinal analysis of female rugby union matches (13). In fact, increases in running intensity and relative collisions were observed and characterized the evolution of female rugby playing style. However, there is a lack of data on the characteristics of competitive French female rugby union and sevens' players. An overview of the evolution of physical characteristics will help coaches adapt their training programs to develop the physical characteristics in accordance with actual requirements.

Focusing on the specificity of practice, during a season, female players may be engaged in both rugby union and rugby sevens championships, and the better players could be selected for the national team. The evolution of French women's rugby performance in international competitions over the last decade supports an improvement, especially in rugby sevens (Table 1). The development of professionalism in rugby sevens since 2014 may explain these improvements. Similar observations have been reported in male rugby union, with players becoming stronger, faster, and fitter in line with the increased professionalism of the game (14, 15). Moreover, sprint performance over all distances was significantly higher in international and professional male players than in club players, especially over the first 10 m (16). Similar results can be found on the 40-m sprint time between professional and amateur male rugby athletes (17).

**Table 1:** List of French sevens and rugby union teams' performances from 2009 to 2020.

	Olympic games (sevens rugby)	Sevens rugby world cup	World rugby sevens’ series	Europe sevens’ grand prix series	Rugby union world cup	6 nations (rugby union)
2009–10		7^th^		5th		4th
2010–11				3rd	4th	2nd
2011–12				5th		2nd
2012–13				3rd		2nd
2013–14		11th	12th	3rd		2nd
2014–15			8th	2nd	3rd	1st (Grand Slam)
2015–16			6th	1st		2nd
2016–17	6th		5th	2nd		1st
2017–18			7th	3rd	3rd	3rd
2018–19		2nd	3rd	2nd		1st (Grand Slam)
2019–20			5th	2nd		3rd
2020–21	2nd		4th			2nd

Therefore, the purpose of this study was to investigate the evolution of anthropometric and physical performances both in French rugby sevens and rugby union over the last decade. We analyzed the evolution of anthropometric and physical performances of French national players over the last decade.

## 2. Materials and Methods

### 2.1. Subjects

Anthropometric and physical performances of 631 French national women rugby union (*n* = 392: 229 forwards and 163 backs; age 25 ± 3 years; height 169.4 cm ± 7 cm; body mass 72.9 ± 12 kg) and rugby sevens' players (*n* = 239; age 24 ± 4 years; height 168.7 cm ± 6.9 cm; body mass 65.7 ± 6.70 kg) were collected from 2009 to 2020. Evaluations were performed two to three times every year, and only the best performance was kept among the evaluations performed every 2 years for each player.

### 2.2. Anthropometric measurements

The height of the players was measured using a height fathom. Body weight was measured using Tanita BC587 scales (Tanita Corp., Tokyo, Japan), and body fat was assessed by dual-beam absorptiometry (D-XA) (Discovery W, Hologic Inc., Marlborough, MA, USA).

### 2.3. Sprint tests

The players completed three 10-m and 20-m tests, and two 50-m sprints on synthetic turf using infrared timing gates (Witty, Bolzano, Italy). The timing gates were placed at hip height, approximately 90 cm above the ground, at 0 m, 10 m, 20 m and 50 m, respectively. The players used rugby cleats and started from a standing position, with their front foot positioned 0.5 m behind the start line. They were instructed to perform all sprints with maximum effort. The best performance was chosen for the analysis.

### 2.4. Maximal aerobic speed

The Yo-Yo IR1 was administered according to the guidelines proposed by Bangsbo et al. (18) and consisted of repeated 2 × 20 m shuttle runs followed by a 10 s active recovery (2 × 5 m of jogging) at a progressively increased speed controlled by an audio signal from a tape recorder until exhaustion. When participants have failed to reach the finish line in time twice, they must stop. Then its total cumulative distance is taken into account, and the maximum aerobic speed is calculated according to the following formula: maximal aerobic speed (MAS) (km h^−1^) = 0.00266 * (Yoyo distance IR1) + 11.51.

### 2.5. Muscular strength

Upper body strength was assessed by an estimated 1 RM test in the bench press followed by the pull-up exercise. In order to standardize the test procedures, the bench press, and the pull-up movements were validated when the bar touched the chest. Free weights were used to perform both tests (Eleiko, Halmstad, Sweden). Several warm-ups set of the exercises were performed with an increase in load each time. When the repetitions were close to 1 RM, participants were allowed to rest for up to 3–5 min before attempting a new load.

### 2.6. Resource statistical analysis

All data are expressed as means ± SD. The Shapiro–Wilk test and Levene's test was applied to verify the normality and homoscedasticity of the variables. A one-way analysis of variance (ANOVA) was used to detect a time effect for each parameter and a one-way ANOVA was used to compare the backs and forwards over time. A Tukey *post hoc* test was used to identify differences when applicable. The significance level was set at *p* ≤ 0.05. A 95% confidence interval (95% CI) was determined with the mean difference, and Cohen's effect sizes (ES) are presented for all variables using thresholds set as negligible (<0.2); small (0.2–0.6); moderate (0.6–1.2); large (1.2–2.0); and very large (>2.0) (19).

## 3. Results

**Figure 1:**
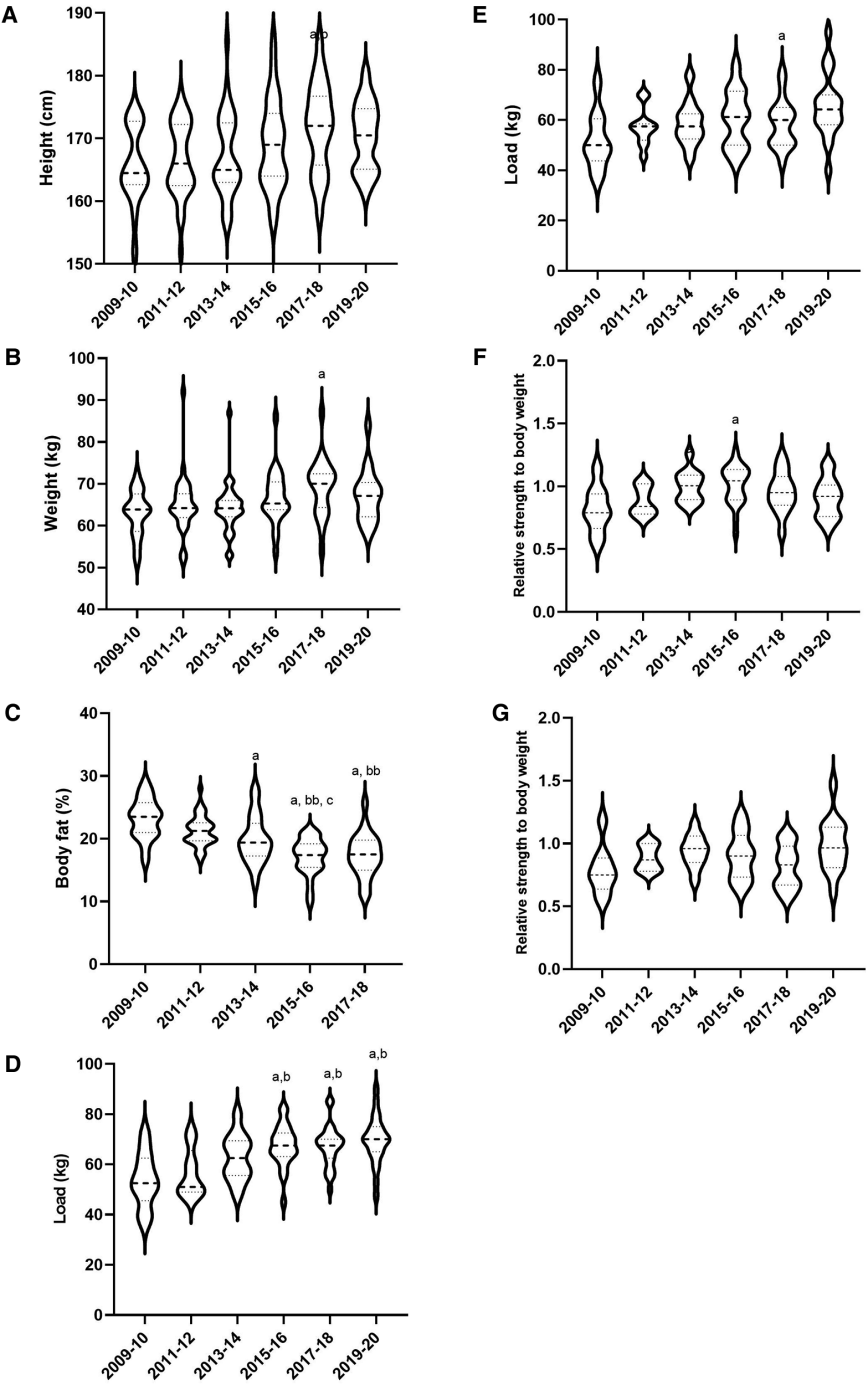
Anthropometric characteristics and physical performance of rugby sevens' players from 2009 to 2020 by 2-year steps. (**A**) Evolution of height, (**B**) Evolution of weight, (**C**) Evolution of body fat, (**D**) Evolution of 1 RM pull, (**E**) Evolution of 1 RM bench press, (**F**) Evolution of 1 RM relative pull, (**G**) Evolution of 1 RM relative bench press. ^a^*p* < 0.05 vs. 2009–10, ^b^*p* < 0.05 vs. 2011–12, ^bb^*p* < 0.01 vs. 2011–12,^c^*p* < 0.05 vs. 2013–14, ^d^*p* < 0.05 vs. 2017–18.

The evolution of anthropometrical and physical performance of rugby sevens' players was presented in Figure 1 and Table 2. Variation of height, weight, and body fat were observed throughout the decade toward a greater height and weight, and a lower body fat percentage. The players exhibited higher strength in pull (Figure 1D) and bench press (Figure 1E) movements in 2020 compared with 2009 with a moderate and large effect (*p* < 0.01 and *p* < 0.05, ES: 1.06 and 1.2, respectively, for pull and bench press). A moderate increase of maximal aerobic speed was observed between 2009 and 2020 (*p* = 0.145 and ES: 0.89, Table 2). Small effects were observed over the sprint performances (Table 2).

**Table 2:** Aerobic capacity and time to 10 m, 20 m, and 50 m comparisons of sevens rugby players.

	2009–10	2011–12	2013–14	2015–16	2017–18	2019–20	Δ 2009–2020	ES 2009–2020
MAS (km·h^−1^)	14.9 ± 0.8	14.9 ± 0.9	15.6 ± 0.9	16.8 ± 1.2^e^^,^^f^^,^^g^	15.9 ± 1^a^^,^^b^^,^^d^	15.6 ± 1.0^h^	+4.5%	0.89
10 m (s)	1.86 ± 0.08	1.87 ± 0.04	1.85 ± 0.05	1.84 ± 0.05	1.80 ± 0.05^a^^,^^b^^,^^c^	1.83 ± 0.08	−1.5%	0.59
20 m (s)	3.23 ± 0.12	3.31 ± 0.12	3.22 ± 0.1	3.19 ± 0.10	3.18 ± 0.11^b^	3.24 ± 0.12	+0.15%	0.20
50 m (s)	7.15 ± 0.37	7.22 ± 0.31	7.06 ± 0.28	6.99 ± 0.30	6.99 ± 0.26	7.08 ± 0.24	−0.9%	0.42

MAS, maximal aerobic speed (km·h^−1^).

^a^ *p* < 0.05 vs. 2009–10,

^b^ *p* < 0.05 vs*.* 2011–12,

^c^ *p* < 0.05 vs. 2013–14,

^d^ *p* < 0.05 vs. 2015–16,

^e^ *p* < 0.01 vs. 2009–10,

^f^ *p* < 0.01 vs. 2011–12,

^g^ *p*<0.01 vs. 2013–14,

^h^ *p* < 0.01. vs. 2015–16.

**Table 3:** Aerobic capacity and time to 10 m, 20 m, and 50 m comparisons of backs rugby union players.

	2009–10	2011–12	2013–14	2015–16	2017–18	2019–20	Δ 2009–2020 (%)	ES 2009–2020
MAS (km·h^−1^)	14.8 ± 0.8	14.3 ± 1.0	15.3 ± 0.8^b^	15.2 ± 1.0^g^	14.8 ± 0.8	15.1 ± 0.9^b^	+2.2	0.64
10 m (s)	1.89 ± 0.06	1.91 ± 0.06	1.89 ± 0.1	1.82 ± 0.08^a^^,^^c^^,^^g^	1.89 ± 0.07^d^	1.87 ± 0.09	−1.1	0.48
20 m (s)	3.33 ± 0.13	3.35 ± 0.09	3.30 ± 0.16	3.19 ± 0.13^b^^,^^c^^,^^f^	3.33 ± 0.13	3.23 ± 0.11^a^^,^^d^	−3.2	0.91
50 m (s)	7.42 ± 0.28	7.42 ± 0.20	7.30 ± 0.38	7.01 ± 0.31^f^^,^^g^^,^^c^	7.34 ± 0.32^h^	7.07 ± 0.26^a^^,^^b^^,^^e^	−4.7	1.11

MAS, maximal aerobic speed (km·h^−1^).

^a^ *p *< 0.05 vs. 2009–10,

^b^ *p *< 0.05 vs*.* 2011–12,

^c^ *p *< 0.05 vs. 2013–14,

^d^ *p *< 0.05 vs. 2015–16,

^e^ *p *< 0.05 vs. 2017–18,

^f^ *p *< 0.01 vs. 2009–10,

^g^ *p *< 0.01 vs. 2011–12,

^h^ *p *< 0.01 vs. 2015–16.

**Table 4:** Aerobic capacity and time to 10 m, 20 m, and 50 m comparisons of forwards rugby union players.

	2009–10	2011–12	2013–14	2015–16	2017–18	2019–20	Δ 2009–2020 (%)	ES 2009–2020
MAS (km·h^−1^)	13.8 ± 1.2	13.5 ± 1.2	14.5 ± 1.0^b^	14.2 ± 1.1^b^	14.4 ± 1.0^b^	14.1 ± 1.1	+2.3	0.52
10 m (s)	1.97 ± 0.1	1.96 ± 0.09	1.98 ± 0.87^a^	1.92 ± 0.09	1.95 ± 0.09	1.92 ± 0.12	−2.7	0.72
20 m (s)	3.51 ± 0.18	3.51 ± 0.15	3.46 ± 0.16	3.35 ± 0.14^b^^,^^c^^,^^d^	3.45 ± 0.17^a^^,^^b^	3.35 ± 0.19^a^^,^^b^	−4.4	0.93
50 m (s)	7.87 ± 0.44	7.78 ± 0.42	7.68 ± 0.41	7.48 ± 0.37^b^^,^^d^	7.67 ± 0.42	7.44 ± 0.46^a^^,^^b^	−5.4	0.99

MAS, maximal aerobic speed (km·h^−1^).

^a^ *p* < 0.05 vs. 2009–10,

^b^ *p* < 0.05 vs. 2011–12,

^c^ *p* < 0.05 vs. 2013–14,

^d^ *p* < 0.01 vs. 2009–10.

**Figure 2:**
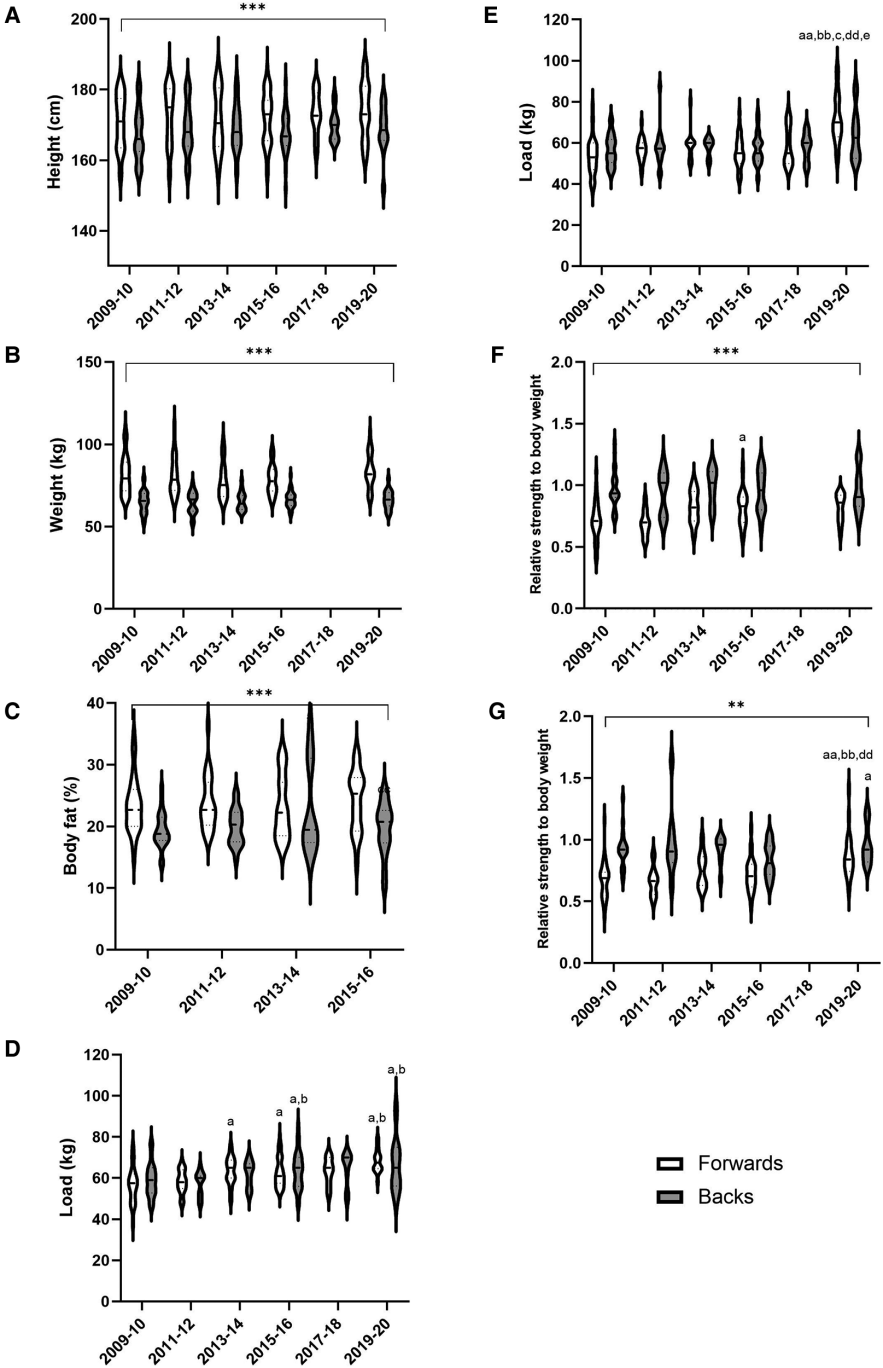
Anthropometric characteristics and physical performance of forwards and backs rugby union players from 2009 to 2020 by 2-year steps. (**A**) Height, (**B**) Weight, (**C**) Fat mass, (**D**) 1 RM pull, (**E**) 1 RM bench press, (**F**) Evolution of 1 RM relative pull, (**G**) Evolution of 1 RM relative bench press. ^a^*p* < 0.05 vs. 2009–10, ^aa^*p* < 0.01 vs. 2009–10, ^b^*p* < 0.05 vs. 2011–12, ^bb^*p* < 0.01 vs. 2011–12, ^c^*p* < 0.05 vs. 2013–14, ^cc^*p* < 0.01 vs. 2013–14, ^dd^*p* < 0.01 vs. 2015–16, ^e^*p* < 0.05 vs. 2017–18, **p < 0.01 forwards vs. backs, ***p < 0.001 forwards vs. backs.

The anthropometric characteristics of rugby union players did not change significantly over the last decade, but differences between forwards and backs were observed (Figures 2A–C). Indeed, forwards were greater and heavier than backs (height: *p* < 0.001, ES: 0.77; weight: *p* < 0.001, ES: 1.55). The rugby union players improved their strength without any difference between forwards and backs (Figures 2D,E). In fact, pull performance increased by 20.6% in the forwards group (*p* < 0.05 and ES: 1.15) and by 12.0% in the backs group (*p* < 0.05 and ES: 0.9, Figure 2D). However, a large increase in bench press performance was observed only the forwards players (*p* < 0.0001 and ES: 1.37, Figure 2E). In addition, a moderate increase of 20 m (*p* < 0.01 and ES: 0.93) and 50 m (*p* < 0.01, ES:0.99) performances in forwards group was observed over the decade. However, no significant difference was observed on 10-m sprint performance and MAS (respectively, *p* < 0.05 and ES: 0.72 for 10-m sprint and *p* < 0.05 and ES: 0.52 for MAS). Similarly, a moderate increase in the 20-m and 50-m sprint was observed in the back group over the decade, with significant variations in years 15–16 and 19–20 compared with the other years, while no effect was observed on maximal aerobic speed and velocity, despite a higher significance in year 15–16 (respectively, *p* < 0.05 and ES: 0.64 for MAS and *p* = 0.05 and ES: 0.48 for 10-m sprint).

## 4. Discussion

The present study described the evolution of the physical and anthropometric characteristics of French female international rugby union and sevens' players over the past 10 years. The evolution differs between rugby union and rugby sevens. While the rugby sevens' players improved mainly their aerobic capacity and their strength, the union players improved both sprint and strength performances. Moreover, anthropometric characteristics of rugby sevens' players evolved toward taller players with less fat mass, whereas no such evolution was observed in rugby union players.

The players were evaluated longitudinally using the same testing battery and testing equipment, which strengthened the present data. The current results are in accordance with previous studies. Indeed, Gabbett (10), Jones et al. (11), and Scantlebury et al. (20) have shown that backs have faster, better aerobic capacity, and a lower percentage of body fat than forwards. These results suggest that specific anthropometric characteristics and physical qualities are required to play a specific position. Furthermore, the specific physical demands of each position will influence the training-induced adaptations. For example, forwards are primarily involved in situations such as tackles, scrums, and battles that require greater body mass, strength, and power (21, 22). On the other hand, backs are involved in high-speed runs, escapes, and movements that require agility and therefore relatively low body mass, but high levels of power and speed (23, 24).

The sprinting performances were better in our study compared with a recent study investigating international rugby woman's performances (20). In fact, the averaged 10-m sprint time was 1.87 vs. 1.88 s in backs and 1.93 vs. 1.99 s in forwards, and the averaged 20-m sprint time was 3.23 vs. 3.28 s in backs and 3.36 vs. 3.45 s The discrepancy may depend on the period of the testing. Indeed, in the study of Scantlebury et al. (20), the players were evaluated during the preseason period, whereas in our study, we retained the best performance on the two or three tests performed by the players during the season. The fluctuations over the decade observed in our study can also be explained by an evolution of training approaches that rely on the coach. Indeed, each trainer has their own convictions about which physical qualities must be developed as a priority. Over the last decade, different coaches drove the national rugby union team while the coach of the rugby sevens' team remained the same. The French Rugby Federation has carried out action plans to develop women's rugby in the country, which has enabled, in an increase in the number of female members and the best players to train more. Thus, most of the rugby academies have been created and/or have become mixed, and the women's clubs have begun to structure themselves more and more. This is mainly characterized by an increase in the number of rugby and strength training sessions, which leads to an increase in the level of young and elite players. Another reason is the increase in professionalism in women's rugby. Since 2014 for women's sevens' players and since 2018 for women's union players, the French Rugby Federation has created professional contracts. This is in line with the study of Scantlebury et al. (20) who observed an improvement in anthropometric and physical qualities alongside the professional development of women's rugby league. This evolution allows players to have access to strength and conditioning coaches who propose structured training sessions compared with non-professional players. These effects can be seen particularly in the rugby sevens' players, who had a peak in performance in 2016 and, following the Olympics, have had a turnover and the transition to a new cycle. And despite the departure of some players, the performance of rugby sevens' players remains better than in 2009.

Regular measurement of physical performance and body composition data over time is an important aspect of monitoring athletes. Such information is useful in making decisions about the magnitude of changes in these variables, and, thus, the success or failure of training. Combining these parameters with the magnitude of the typical error of each test would allow the identification of changes that are significant for the players (25). For example, the typical error (as a coefficient of variation) for the L1 Yo-Yo IRT in the rugby players in this study indicated that intermittent running performance varied by approximately 10.9% from test to test when performed 1–2 months apart. Adding this reliability to the smallest change observed in other variables (skinfold thickness, body mass, 10-m, 20-m, and 50-m sprint) suggests that there is too much variation in performance to identify valid smallest effect changes and that only larger effect sizes will be noticed until measures to reduce test to test variation are implemented (25). The combination of these parameters with the magnitude of the typical error of each test would allow us to identify changes that are significant for the players. For example, the typical error (as a coefficient of variation) for a 30-m sprint is less than 1.7% in amateur rugby player (26). The developments observed in our study suggest that rugby union players have improved their sprinting ability over the last decade. Despite a slight increase in 20-m time, female sevens' players have also improved their sprinting ability (0.3 s over 10-m sprint and 0.3 s over 50-m sprint) over the last decade.

Identifying the physical and anthropometric performance characteristics required to play at the international level would help in recruiting and developing players. Indeed, one of the growing pressures on sports organizations is to identify promising young athletes and provide them with an optimal learning environment to facilitate long-term performance (27). Some physical variables appear to discriminate in talent detection. For example, Till et al. highlighted the importance of anthropometric and physical qualities (height and body mass, body fat percentage, linear speed, change-of-direction speed, aerobic capacity, strength, muscular low power) for the identification and development of young rugby league players (28). The present results support the notion that the evaluation of anthropometric parameters, sprint, and strength performances will help to discriminate players and that the improvements observed over the last decade rely on the same parameters used for talent identification. These parameters are considered important factors to differentiate players according to their age group, level of competition, and position (29, 30). A recent study suggests that those selected from the Regional Academy were significantly heavier, stronger, and faster over 20 m compared with their unselected peers, with effect sizes for anthropometric, physiological, and cognitive factors ranging from small to large (30). The fluctuations may be explained by the international event program. Indeed, we could expect that the best athletes reach their peak performance the year of a major event such as the World Cup or the Olympic Games. After the event, some of the best players stopped their careers and were replaced by young players characterized by lower performances. Moreover, most of the coaches periodize the development of quality over a 4-year cycle. Overall, these results suggest that physical and anthropometric variables appear to be key elements in identifying talent and having the potential to reach the level of expertise in a particular sport, as suggested by Williams and Reilly (31).

The testing sessions were organized without taking into consideration the menstrual cycle. Indeed, Antero et al. (32) highlight variations in performance between the periods of the menstrual cycle and differences in performance between players who have contraception compared with those who do not. We, therefore, suggest that future studies should consider the timing of the menstrual cycle to avoid misleading results or the lack of a training-induced effect.

## 5. Practical applications

The description of the evolution of anthropometric and physical performance and their current values is important as it will help identify the characteristics of the international squad and to help select the players. Indeed, the players are required to have well-developed anthropometric characteristics and physical performances to perform at the highest level possible (7). A regular assessment of the physical performances is useful for developing an adapted strength and conditioning program to prepare players for the rugby-specific demands (33).

## Data Availability

The raw data supporting the conclusions of this article will be made available by the authors, without undue reservation.

## References

[1] Sarthou J-J. Enseigner le rugby en milieu scolaire, (2006), Les Cahiers Actio.

[2] Hendricks S Karpul D Lambert M. Momentum and kinetic energy before the tackle in rugby union. J Sports Sci Med. (2014) 13:557–63. PMID: ; PMCID: . 25177182 PMC4126292

[3] Hendricks S Lambert M. Tackling in rugby: coaching strategies for effective technique and injury prevention. Int J Sports Sci Coach. (2010) 5(1):117–35. 10.1260/1747-9541.5.1.117

[4] Hendricks S Sin DW van Niekerk T den Hollander S Brown J Maree W Technical determinants of tackle and ruck performance in international rugby sevens. Eur J Sport Sci. (2019) 20:1–27. 10.1080/17461391.2019.1675764 31665980

[5] Ross A Gill N Cronin J. Match analysis and player characteristics in rugby sevens. Sports Med. (2013) 44:357–67. 10.1007/s40279-013-0123-0 24234931

[6] Cummins C Melinz J King D Sanctuary C Murphy A. Call to action: a collaborative framework to better support female rugby league players. Br J Sports Med. (2020) 54:501–2. 10.1136/bjsports-2019-101403 31996346

[7] Emmonds S Heyward O Jones B. The challenge of applying and undertaking research in female sport. Sports Med Open. (2019) 5(1):51. 10.1186/s40798-019-0224-x 31832880 PMC6908527

[8] Dobbin N Highton J Moss SL Twist C. The discriminant validity of a standardized testing battery and its ability to differentiate anthropometric and physical characteristics between youth, academy, and senior professional rugby league players. Int J Sports Physiol Perform. (2019) 14:1110–6. 10.1123/ijspp.2018-0519 30702356

[9] Tredrea M Dascombe B Sanctuary CE Scanlan AT. The role of anthropometric, performance and psychological attributes in predicting selection into an elite development programme in older adolescent rugby league players. J Sports Sci. (2017) 35:1897–903. 10.1080/02640414.2016.1241418 27724178

[10] Gabbett TJ. Physiological and anthropometric characteristics of elite women rugby league players. J Strength Cond Res. (2007) 21:875–81. 10.1519/R-20466.1 17685702

[11] Jones B Emmonds S Hind K Nicholson G Rutherford Z Till K. Physical qualities of international female rugby league players by playing position. J Strength Cond Res. (2016) 30:1333–40. 10.1519/JSC.0000000000001225 26439784

[12] Yao X Curtis C Turner A Bishop C Austerberry A Chavda S. Anthropometric profiles and physical characteristics in competitive female English premiership rugby union players. Int J Sports Physiol Perform. (2021) 16(9):1234–41. 10.1123/ijspp.2020-0017 33626507

[13] Woodhouse LN Tallent J Patterson SD Waldron M. International female rugby union players' anthropometric and physical performance characteristics: a five-year longitudinal analysis by individual positional groups. J Sports Sci. (2022) 40(4):370–8. 10.1080/02640414.2021.1993656 34706619

[14] Lombard W Durandt J Masimla H Green M Lambert M. Changes in body size and physical characteristics of South African under-20 rugby union players over a 13-year period. J Strength Cond Res. (2015) 29:980–8. 10.1519/JSC.0000000000000724 25387267

[15] Haugen TA Tønnessen E Seiler S. Speed and countermovement-jump characteristics of elite female soccer players, 1995–2010. Int J Sports Physiol Perform. (2012) 7:340–9. 10.1123/ijspp.7.4.340 22645175

[16] Watkins CM Storey A McGuigan MR Downes P Gill ND. Horizontal force-velocity-power profiling of rugby players: a cross-sectional analysis of competition-level and position-specific movement demands. J Strength Cond Res. (2021) 35(6):1576–85. 10.1519/JSC.0000000000004027 33927113

[17] Ross A Gill ND Cronin JB. Comparison of the anthropometric and physical characteristics of international and provincial rugby sevens players. Int J Sports Physiol Perform. (2015) 10(6):780–5. 10.1123/ijspp.2014-0331 25229160

[18] Bangsbo J Iaia FM Krustrup P. The Yo-Yo intermittent recovery test: a useful tool for evaluation of physical performance in intermittent sports. Sports Med. (2008) 38:37–51. 10.2165/00007256-200838010-00004 18081366

[19] Hopkins WG Marshall SW Batterham AM Hanin J. Progressive statistics for studies in sports medicine and exercise science. Med Sci Sports Exerc. (2009) 41(1):3–13. 10.1249/MSS.0b013e31818cb278 19092709

[20] Scantlebury S McCormack S Sawczuk T Emmonds S Collins N Beech J The anthropometric and physical qualities of women's rugby league super league and international players; identifying differences in playing position and level. PLoS One. (2022) 17(1):e0249803. 10.1371/journal.pone.0249803 PMC8803183 35100275

[21] Cummins C Orr R H. O’Connor C. West Global Positioning Systems (GPS) and microtechnology sensors in team sports: a systematic review. Sports Med. (2013) 43:1025–42. 10.1007/s40279-013-0069-2 23812857

[22] Coughlan GF Green BS Pook PT Toolan E O’connor SP. Physical game demands in elite rugby union: a global positioning system analysis and possible implications for rehabilitation. J Orthop Sports Phys Ther. (2011) 41:600–5. 10.2519/jospt.2011.3508 21654094

[23] King D Cummins C Hume PA Clark TN Pearce AJ. Physical demands of amateur senior domestic rugby union players over one round of competition matches in New Zealand assessed using heart rate and movement analysis. Int J Sports Sci Med. (2018) 2:66–71. http://hdl.handle.net/10292/12108.

[24] Takamori S Hamlin M Kieser D King D Hume P Yamazaki T Senior club-level rugby union player's positional movement performance using individualized velocity thresholds and accelerometer-derived impacts in matches. J Strength Condit Res. (2020) 36(3):710–6. 10.1519/JSC.0000000000003523 32168074

[25] Hamlin MJ Deuchrass RW Elliot CE Manimmanakorn N. Short and long-term differences in anthropometric characteristics and physical performance between male rugby players that became professional or remained amateur. J Exerc Sci Fit. (2021) 19(3):143–9. 10.1016/j.jesf.2021.01.002 33680002 PMC7895839

[26] Zabaloy S Freitas TT Carlos-Vivas J Giráldez JC Loturco I Pareja-Blanco F Estimation of maximum sprinting speed with timing gates: greater accuracy of 5-m split times compared to 10-m splits. Sports Biomech. (2021):1–11. 10.1080/14763141.2020.1838603 33428549

[27] Baker J Cobley S Schorer J Wattie N. Talent identification and development in sport. In: Backer J Cobley S Schorer J Wattie N, editors. Routledge handbook of talent identification and development in sport. London: Routledge (2013). p. 1–7.

[28] Till K Scantlebury S Jones B. Anthropometric and physical qualities of elite male youth rugby league players. Sports Med. (2017) 47(11):2171–86. 10.1007/s40279-017-0745-8 28578541 PMC5633637

[29] Owen C Till K Weakley J Jones B. Testing methods and physical qualities of male age grade rugby union players: a systematic review. PLoS One. (2020) 15:e0233796. 10.1371/journal.pone.0233796 32497130 PMC7272054

[30] Dimundo F Cole M Blagrove RC McAuley ABT Till K Kelly AL. Talent identification in an English premiership rugby union academy: multidisciplinary characteristics of selected and non-selected male under-15 players. Front Sports Act Living. (2021) 3:688143. 10.3389/fspor.2021.688143 34179777 PMC8225930

[31] Williams AM Reilly T. Talent identification and development in soccer. J Sports Sci. (2000) 18:657–67. 10.1080/02640410050120041 11043892

[32] Antero J Golovkine S Niffoi L Meignié A Chassard T Delarochelambert Q Menstrual cycle and hormonal contraceptive phases' effect on elite rowers' training, performance and wellness. Front Physiol. (2023) 14:220. 10.3389/fphys.2023.1110526 PMC9981658 36875020

[33] Gabbett TJ Stein JG Kemp JG Lorenzen C. Relationship between tests of physical qualities and physical match performance in elite rugby league players. J Strength Cond Res. (2013) 27(6):1539–45. 10.1519/JSC.0b013e318274f236 23037614

